# Hospital Finance

**Published:** 1923-02

**Authors:** 


### HOSPITAL FINANCE.

### THE RESULT OF THE COMBINED APPEAL.

AT the time of writing the total received from the Hospitals of London Combined Appeal, which is now concluded, is over £460,000 ; Mr. Shrapnell-Smith, the director-general, has completed his task, and outstanding matters, which include receipts still to be tabulated, are in charge of Mr. F. B. Elliot, C.B.E., the Secretary of the Appeal Committee. The General Election hindered matters somewhat, but the desired total of half a million is believed to have been raised, inclusive of the proceeds of the prize competition. On another page our readers have the benefit of the Hon. Sir Arthur Stanley's verdict upon the campaign, which represents his considered opinions of what, after all, has been a great experiment, as they have impressed themselves upon him at headquarters. Complete unanimity could hardly be expected among individual hospitals participating, since some were reluctant to adopt the plan and others believe, with Mr. E. W. Morris, of the London Hospital, that they have received, in the past, from their individual efforts, more than falls to their share from the Combined Appeal. But the verdict is not entirely a matter of figures. We have to ask ourselves if the experiment is to be repeated, and, if so, whether its repetition is likely to produce larger or smaller results on a subsequent occasion. It may be argued that, in London, hospital co-operation is most valuable outside the sphere of appeals ; that the King's Fund already provides a common purse, and that the combination of hospitals in services such as are offered by the Hospitals Saving Association is the best common ground, and the most feasible method of raising an annual revenue. The questions have ceased to be abstract since the experiment of a Combined Appeal has been made. It was worth making, and that its lessons may be learnt we place side by side one opinion from headquarters and one from a participating hospital.

### HOSPITALS AND THE UNEMPLOYED.

KING EDWARD'S Hospital Fund for London has sent to the Government and to the Voluntary Hospitals Commission an important memorandum suggesting that schemes for hospital extensions, alterations and repairs should be favourably considered when public funds are allotted for the provision of work for the unemployed. The grants made by the Fund in aid of such schemes this year amounted to £26,900. Prior to the Fund's decision to send this memorandum, Lord Northampton, Chairman of the Royal Northern Hospital, had already urged that the Government should assist by grants or loans similar building schemes through the unemployment grants committee. Lord Northampton's proposal was despatched to the Ministry of Labour and the Ministry of Health, and in the case of his hospital £24,000 out of the £35,000 required is already in hand. The building of a new casualty department and a new nurses' home would provide much work for the unemployed in North London, and the same, of course, is true all over the country. It is generally admitted that there is still a serious scarcity of hospital beds, and consequently the assistance proposed would give not only help to the unemployed during the construction, but to the men and their families in times of sickness. Fewer objections can be urged against this proposal than against many of the schemes which the prevalence of unemployment has called forth, and, since such grants would be given upon industrial grounds, the Government would not be interfering with the voluntary system, but helping the hospitals indirectly in furtherance of a general policy to which it is already pledged. In these circumstances we hope that the King's Fund memorandum will be sympathetically considered to the mutual benefit of the hospitals and of the unemployed.

### SIR NAPIER BURNETT AT BRIGHTON.

SIR NAPIER BURNETT, Director of Hospital Services, has personally investigated the affairs of the Royal Sussex County Hospital, Brighton, the financial condition of which led to the appointment of a Committee of Inquiry last autumn. Sir Napier's report has now been presented to the Committee. Its recommendations will be read with added interest, since much was hoped from the so-called Sussex scheme for organised contributions associated with the institution. But for reasons that are not explained the Committee apparently proposes not to publish Sir Napier's report or conclusions, but only the result of its deliberations upon them. Foreseeing doubtless that this attitude might be misunderstood, the Committee declares that there is nothing in the report "to deter the public 'from continuing its support.'" This being so, to withhold it is an error of tactics. The report, or at least its conclusions, since it is understood to be voluminous, would have excited general interest, and perhaps done more than anything else to focus public attention upon the needs of the hospital. Mr. Roden Orde was, we believe, assisting Sir Napier in his inquiry, and the opinions and suggestions of two such authorities would have been of general value. It is also hardly in accord with voluntary hospital practice to withhold such documents.

### THE FATE OF THE LISTER WARD.

THE future of the famous Lister Ward in the Glasgow Royal Infirmary has been again the subject of discussion. In his recent visit, the Lord Provost, Sir Thomas Paxton, Bt., after referring to the opening of the home for retired nurses at Dumbreck, said that he had always been one of those in favour of the preservation of the Lister Ward, the decision to demolish which he hoped that the managers of the Royal Infirmary would reconsider. It was on the ground floor of the old block, and if the upper storeys were taken away he thought the Lister

Ward would not present any unsightly obstruction to the present building. Mr. James Macfarlane, chairman of the managers, who followed, said that if the boundaries of the Infirmary could be extended, there would be no difficulty in retaining the ward, which was on the ground required. If it could be removed and rebuilt on some other suitable site, his colleagues would give every facility. He believed that if Lord Lister were alive, he would support the managers' view. On this one may remark that no more specious argument could be imagined, for what man could place the perpetuation of his work and memory before the immediate convenience of the institution where it was performed? No man can be a judge in his own case, and it is reprehensible to pretend to consult Lister's wishes in such a matter. The Lister Ward is an historic building, the most famous ward in the world, and, like all such monuments, it is held in trust by each generation for posterity. In the absence of sufficient public spirit to provide an alternative site, it may be impossible to save the ward; but for the managers to destroy it, and to take credit for so doing, is ludicrous. Such an act can be a regrettable necessity at best, and one which later generations will find it harder to forgive than our own.

---

### HOSPITAL POSTS AND LOCAL CANDIDATES.

A NUMBER of medical practitioners in Hastings have protested against the appointment of a surgeon non-resident in the district to the staff of the East Sussex Hospital. They criticise the appointment on the grounds that it will tend to keep medical talent from the town, to diminish the interest of the profession in the hospital, and that it is opposed to the policy of the Ministry of Health. The signatories also express their regret that the staff of the hospital did not protest against the appointment of a non-resident candidate. This, surely, much weakens their argument. Is it not the fact that hospital committees are free to appoint the best candidate, in their judgment, who appears? And were appointments limited in practice to local medical men, would not their advertisement be a farce, since other candidates would feel it useless to reply to them ?

---

### REGULAR CONTRIBUTIONS IN EDINBURGH.

IT is a significant example of the value of organised weekly contributions that, despite unemployment and a net decrease of £2,640 in the returns of 440 firms, the total membership of the League of Subscribers connected with Edinburgh Royal Infirmary rose to 85,000, an increase of 10,000 members, during the past year. The grand total subscribed by this means was therefore more than £500 to the good, a result chiefly encouraging in the prospects that it affords when better times return. The accounts show a deficit of £20,000 upon the ordinary expenditure, for if the economies amounted to £13,500, the ordinary income shrunk, as we see, even more. Legacies and other extraordinary income amounted to £80,000; so the hospital has survived a difficult year with cause for relief.

